# Editor’s Book Table

**Published:** 1871-07

**Authors:** 


### Editor's Book Table.

[NOTE.—All works reviewed in the columns of the CHICAGO MEDICAL JOURNAL may be found in the extensive stock of W. B. KEEN & COOKE, whose catalogue of Medical Books will be sent to any address upon request.]

*A Practical Treatise on the Diseases of Infancy and Childhood.* By THOMAS H. TANNER, M.D., F.L.S. Third American Edition. From the last London Edition. Revised and Enlarged, by ALFRED MEADOWS, M.D., London. Philadelphia: Lindsay & Blakiston. 1871.

The author of the two preceding editions of this popular work, being unable from a press of business engagements to superintend the revision of this third, has confided the task to an able colleague, and we have the result of his learning and energy in the shape of one of the best books ever written on the diseases of children.

The division into four parts, strikes us as being particularly happy; affording a much more systematic arrangement, and a wider field for the intelligent discussion and understanding of this most difficult and comprehensive branch of medicine.

In the *first* part, he gives us as the first step, a clear statement of the anatomical and physiological peculiarities of childhood, to a thorough knowledge of the various pathological conditions so different as found in the adult, and then proceeds to discuss the hygienic management, embracing the dietary, clothing, exercise, sleep, moral and intellectual training, from the first moment of life to the dawning of maturity. The chapter on dentition should be carefully studied, as affording a solution of the various processes attending the eruption of the deciduous and permanent teeth, and the disorders consequent thereon; and next, the pathology of infancy and childhood, with the statistics of mortality. It cannot be too strongly impressed upon the minds of medical men, the importance of a systematic study, and a proper interpretation of the symptomatology of these diseases, from the expression of the countenance, the gestures and attitudes, the examination of every organ, to a chemical analysis of the urine, if necessary; and it will be only by years of close and accurate observation, that we can hope to be successful in combating the various ailments brought to our observation.

It seems almost superfluous to state that, from the entrance into the sick room, and the first step in our examination,—the taking of the pulse,—to the last,—the inspection of the throat, mouth and gums, (the grievous part of the medical visit, as Dr. West terms it), the most untiring patience, and unvarying good humor, with quiet demeanor and gentle voice, are absolutely necessary to succeed at all, and to those testy and choleric individuals, who cannot endure the wail of an infant, or the fractious yelps of a spoiled child, without a hankering to apply their palms *à posteriori*, or throw them out of the window, we would recommend that their energies be devoted to other branches, for as our author says, "it is by no means easy to understand a helpless infant; his only language is a language of signs, which nothing but habit, experience and patience will enable us to interpret."

The last chapter of this part is devoted to infantile therapeutics, and is deserving of close attention. The proper manner of using and combining, the physiological action of the various remedies, and the conditions calling for their employment, are succinctly set forth, and we the more readily endorse his views, because he leans to the side of conservatism, and does not believe that we possess any of those specific receipts, which so many of our physicians carry in their heads, or if short of memory, in their pocket-books. He believes, and justly too, that many diseases may be avoided by attention to food, cleanliness, and the ordinary rules of hygiene, and does not believe that indiscriminate and hap-hazard medication is at all called for; yet, at the same time, admits the necessity, under certain circumstances, of heroic treatment, inasmuch as acute and destructive disorders run a much more rapid course, and terminate in dissolution more certainly than in the adult. We think this chapter one of the most interesting and instructive in the whole book, and therefore commend it to all, especially the young practitioner.

In the *second* division, he treats of general diseases,—the varieties of fevers, and diathetic disorders. His classification of fevers expresses a truer pathology than any we have seen; the *continued* fevers, typhoid and typhus; the *intermittent* fevers; and the *eruptive* fevers, disregarding entirely those symptomatic, or irritative pyrexial manifestations, arising from inflammations, incidental to other disorders, and merely considering those in which the group of symptoms constitute, what we call fever,—the very essence of the disease. He tells us of *four* diatheses, hereditary or acquired: the scrofulous; the tubercular; the rachitic; and the syphilitic. They possess some few features in common, but are entirely distinctive, and in many respects antagonistic. For instance, the scrofulous is entirely distinct from the tubercular, having nothing in common, if we except debility, or depraved health, and never occurring in the same subject. The pathological tendencies of the former are exhibited by inflammation of low type, with marked disposition to suppuration, and this with special reference to mucous surfaces, pulmonary and gastro-intestinal; whereas, in the tubercular, the tendency is towards serous membranes, with effusion. In the former besides, glandular enlargements, in the latter tuberculous deposits, and they are as essentially different in many other particulars, as small-pox from scarlet fever, or measles. This runs counter to the opinion of many practitioners, whom we know personally, and who always consider the tuberculous as one of the phases of the scrofulous constitution, but at this present stage of our pathology, they are recognized as possessing opposite and diverse characteristics.

The rachitic is also a disease of mal-nutrition, and affects principally, but by no means entirely, the osseous structures, and sometimes, though rarely, may be engrafted on the strumous.

The syphilitic diathesis of course may be found combined with any of the preceding, and is a disease *sui generis*, either hereditary or acquired. It can be transmitted from either parent, without doubt, and is capable of spreading infection likewise.

The influence which these diatheses have upon the various diseases of younger life, is of course incalculable; the characters which they impress; the pathological tendencies they exhibit; and the therapeutical indications they demand, can only be estimated by their bearings upon present and future generations; and especially is this the case, when we know that such dreadful scourges may be inflicted upon perfectly sound and healthy children, by deprivation from light, heat, nourishing food, and lastly, —*sub rosâ* be it said,—by unsound medication, creating debility and prostration, sapping the very springs of life, by such remedies as antimony, calomel, blood-letting, etc., pushed *usque ad mortem.*

Part *third* opens with the nervous diseases, a special class, more obscure, and more terrible in their manifestations than any other in the category, besides presenting a high rate of mortality. On account of the rapid development of the brain, and the activity of the circulation in early childhood, almost every disease is ushered in, or accompanied by decided, and sometimes alarming nervous phenomena, and it becomes necessary to understand the symptoms of nervous disorders, functional or organic, if we hope for success in the treatment of children. In the chapter on respiratory diseases, we find an excellent division, beginning with the nasal passages, throat, etc.; next, larynx and bronchial tubes; then, serous surfaces; and lastly, lung tissue itself. The mortality, absolutely speaking, is greater in these disorders of the respiratory system, than those of the nervous, but relatively, it is not so, from their greater frequency, and probably there is no class of diseases more commonly associated with severe disturbance of the nervous centres than those discussed above.

Of those sections, treating of the circulatory and urinary systems, we have but little to say, except that the arrangement is unexceptionable, the pathology sound, and up to the most modern investigations. And the treatment, as in that of the pulmonary ailments, essentially scientific, sustaining, and expectant, never rash, lowering nor heroic.

The classification of skin diseases in chapter fourth, is the work of Dr. Tilbury Fox, one of the most learned and successful dermatologists in England. It is much simplified, and divested of many technical terms, for a better understanding by the younger practitioner, and presents the treatment and diagnosis in a clear and unmistakable light. As these cutaneous diseases are very rife among children, and as a rule are readily curable, provided their pathology be fairly understood, and their causation too, we hope every one will give this section an especial consideration. The next two chapters treat of diseases of the eye and ear, and like the one on those of the skin, are well placed before the reader, but they present simply the outlines of the various diseases which afflict these important organs, and can only be considered as guides to a proper study of the monographs. Without a thorough knowledge, and extensive experience, if severely visited, we had better send them to those who make a specialty of such branches.

In part *fourth*, and the last, the many accidents, injuries and deformities at and after birth are brought before us, and though this properly belongs to the domain of surgery, and in some few particulars to midwifery, yet we must look upon it as a very useful and handsome supplement to complete this instructive work.

In the appendix, we encounter a large collection of formulæ and receipts for making the necessary articles of diet, etc., to be used in the sick room, and also a large number of scientific prescriptions to meet the morbid conditions of the many diseases of children. We should hardly finish a conscientious review of this book, did we not offer a caution, especially to the new beginner, that in looking over these combinations, he should do so merely with the view of learning how remedies are combined, those which are compatible, their doses, and the indications calling for such uses: but the moment he shall attempt to commit them to memory, he had better close the book at once. No especial remedy or combination will suit persons of different temperaments, and under the infinitely varying conditions of the human system, and no stereotyped prescriptions will be dealt out to Tom, Dick and Harry, as was the treacle and sulphur to the squalid children of "Dotheboy's Hall," by any scientific physician, who reasons out the requirements of each case, and then applies his knowledge. Any other way is empirical, and if you once begin thus, you will necessarily degenerate, for the road to true knowledge is no golden one, and the "imp of idleness" ever stands at our elbow to draw us from the path of duty. Go into any drug store, and study his prescriptions, if you are properly instructed, you will read your man at once, for "ye shall be known by your works."

To finish, we endorse this book heartily; it is equal to West, and Barthez, and Rilliet; embodies a vast amount of information; is very clear and intelligible in its descriptions; and will certainly meet the requirements of all desiring to make study of the diseases of infancy and childhood.

"VOLTA REEKE."

BOOKS RECEIVED.

*Treatise on Diseases of the Nervous System.* By WILLIAM A. HAMMOND, M.D., Prof. of Diseases of the Mind and Nervous System, and of Clinical Medicine in Bellevue Hospital Medical Coll., etc., etc. With Forty-five Illustrations. *Est quoddam prodire tenus, si non datur ultra.*—HOR. New York: D. Appleton & Co., 549 and 551 Broadway. 1871. Pp. 754. By S. C. Griggs & Co., Chicago.

*The Eye in Health and Disease.* By B. JOY JEFFRIES, A.M., M D., Fellow of the Massachusetts Medical Society, Member of the American Ophthalmological Society, Ophthalmic Surgeon to the Massachusetts Charitable Eye and Ear Infirmary, Ophthalmic Surgeon to the Carney Hospital, Lecturer on Optical Phenomena and the Eye, at Harvard University. CONTENTS—Anatomy of the Eye. Physiology of the Eye. Old Sight and Spectacles. Near-Sightedness, or Myopia. Long-Sightedness, or Over-Sightedness—Hypermetropia. Astigmatism. Cataract in Children simulating Near-Sightedness. Cataract. Artificial Eyes—How and Why they are Worn. Squinting Eyes—Why and How they must be operated on. An Artificial Pupil—What it is, How and Why the Operation is performed. The Ophthalmoscope—What it is, and How it is used. Injuries and Diseases of the Lids and Eye—Their General Care and Treatment. Type for Testing Vision. With 30 illustrations, 8vo, cloth. Price, $1.50, mailed free, on receipt of price. Alexander Moore, Publisher & Bookseller, 2 Hamilton Place, Boston.

*On the Physiological Effects of Severe and Protracted Muscular Exercise. With special reference to its Influence upon the Excretion of Nitrogen.* By AUSTIN FLINT, JR M.D., Prof. Physiology in the Bellevue Hospital Med. Coll., N. Y., etc., etc. New York: D. Appleton & Company, 549 and 551 Broadway. 1871. Pp. 91.

This is a scientific account of the physiological phenomena observed during the time occupied by the celebrated pedestrian, Weston, in accomplishing the feat of walking 100 miles in twenty-one hours and thirty-nine minutes, and subsequently, in several similar prolonged muscular efforts by the same person. The general account and summing up are given by Prof. Flint in his usual careful and lucid manner.

PAMPHLETS.

*A Month's Tour through the Alps of Switzerland.* By PROF. JAMES D. DANA, LL.D., Yale College, etc., New Haven, Conn. Charles C. Chatfield & Co., New Haven, Conn. Pp. 11. S. C. Griggs & Co. 25c.

Convenient for reference to the traveler, as indicating the route for both the savan and the sight seer. A detail of expenses is given from New York and return.

*The Detection of Criminal Abortion.* By ELY VAN DE WARKER, M.D., Syracuse, N. Y. Boston: James Campbell, Publisher, 18 Tremont St., Museum Building. 1871. Pp. 16.

